# Platelet-rich plasma for wound healing and scar formation after cesarean section: a systematic review and meta-analysis of randomized controlled trials

**DOI:** 10.1186/s12884-026-09447-w

**Published:** 2026-06-11

**Authors:** Serhat Şen, Feyza Gülgel Şen

**Affiliations:** 1 Department of Gynecologic Oncology, Liv Topkapı Hospital, İstinye University, Istanbul, Turkey; 2 Department of Obstetrics and Gynecology, Medical Park Gaziosmanpaşa Hospital, İstinye University, Istanbul, Turkey

**Keywords:** Platelet-rich plasma, Cesarean section, Wound healing, Scar, Uterine niche, Isthmocele, POSAS, REEDA, Meta-analysis, Systematic review

## Abstract

**Background:**

Surgical wound complications follow 3–15% of cesarean deliveries globally. Platelet-rich plasma (PRP), an autologous concentrate of growth factors, has documented regenerative activity across several surgical disciplines, yet no quantitative synthesis of randomized trial evidence has been published for its intraoperative use during cesarean section.

**Methods:**

PubMed/MEDLINE, Embase, CENTRAL, Scopus, and Web of Science were systematically searched from inception through March 2026 to identify randomized controlled trials comparing intraoperative PRP with standard care during cesarean section. Primary outcomes were skin scar quality measured with the Patient and Observer Scar Assessment Scale (POSAS), the Vancouver Scar Scale (VSS), or the Manchester Scar Scale; early wound inflammation measured with the Redness, Edema, Ecchymosis, Discharge, and Approximation (REEDA) scale; and uterine scar integrity defined by niche formation rate and residual myometrial thickness. Secondary outcomes were postoperative pain on visual analogue or numeric rating scales and analgesic consumption. Standardized mean differences (Hedges’ g) were pooled using a DerSimonian–Laird random-effects model. Ambiguity regarding dispersion measures in one study was resolved through p-value back-calculation, and sensitivity analyses were pre-specified. The protocol was registered prospectively (INPLASY202640041; doi: 10.37766/inplasy2026.4.0041).

**Results:**

Five randomized trials enrolling 366 women across five countries were included, with four trials (*n* = 336) contributing to skin-scar synthesis. PRP improved patient-reported scar quality (POSAS-patient SMD − 0.50, 95% CI − 0.83 to − 0.17; *p* = 0.003; I² = 0%) and clinician-assessed scar quality (POSAS-observer SMD − 0.42, 95% CI − 0.75 to − 0.10; *p* = 0.012; I² = 32%), and reduced early wound inflammation (REEDA SMD − 0.52, 95% CI − 0.84 to − 0.20; *p* = 0.002; I² = 0%; sensitivity analysis under the naive SD assumption produced I² = 98% and was uninterpretable). In a single small trial that targeted the uterine scar, PRP reduced niche formation from 46.7% to 13.3% (RR 0.29, 95% CI 0.07–1.16; Fisher’s exact *p* = 0.109 on the binary 2 × 2 table). Vancouver Scar Scale heterogeneity precluded pooling (I² = 94%). Visual analogue pain at early time points was not significantly reduced (pooled SMD − 0.22, 95% CI − 0.50 to + 0.06; *p* = 0.12). No safety event attributable to PRP was reported in any trial.

**Conclusions:**

Across a small and clinically heterogeneous evidence base of five randomized trials, intraoperative PRP improves cesarean skin scar quality and reduces early wound inflammation, while early pain intensity does not appear to be affected. The single trial that addressed the uterine scar suggests a substantial reduction in niche formation, but with only thirty patients, an unreproducible study-reported p-value, and a Fisher’s exact p of 0.109 on the binary outcome, this signal is hypothesis-generating and requires confirmation in adequately powered trials before any change in practice can be considered. Protocol registration: INPLASY202640041 (doi: 10.37766/inplasy2026.4.0041).

**Supplementary Information:**

The online version contains supplementary material available at 10.1186/s12884-026-09447-w.

## Background

Cesarean section accounts for approximately 21% of all births worldwide, a proportion projected to reach 28.5% by 2030 [1]. Wound complications, including infection, dehiscence, seroma, and hematoma, affect between 3% and 15% of patients, and substantially more in high-risk groups [2, 3]. Women with class III obesity carry a two- to fourfold higher risk of wound breakdown, and women undergoing repeat cesareans operate through progressively less well-vascularized scar tissue. Enhanced Recovery After Surgery (ERAS) protocols have reduced complications through prophylactic antibiotics, skin antisepsis, and subcuticular closure, yet the residual complication rate is not zero [4], and the populations at highest absolute risk are precisely those most consistently excluded from the randomized trials that generate the evidence base.

Platelet-rich plasma is derived from a small volume of the patient’s own blood by centrifugation, yielding a fraction enriched two- to fivefold in platelets. On activation, platelets release platelet-derived growth factor, transforming growth factor-β, vascular endothelial growth factor, epidermal growth factor, fibroblast growth factor, and insulin-like growth factors 1 and 2 in a coordinated surge that shortens the inflammatory phase, drives fibroblast proliferation and collagen synthesis, and supports angiogenesis in granulation tissue [5, 6]. The biological rationale for PRP at the cesarean incision parallels its rationale at the tendon, the diabetic foot, and the cardiac wound: an injured tissue in which growth-factor-mediated repair is the limiting step is plausibly accelerated by delivery of an autologous concentrate at the wound margins. Cord blood PRP (CB-PRP), collected from the umbilical vessels after delivery, is uniquely available only at cesarean section and is reported to deliver a growth-factor payload that exceeds adult peripheral blood by virtue of fetal haematopoietic biology [7].

One aspect of the cesarean wound has been almost entirely absent from the meta-analytic literature: the uterine scar itself. Between one-third and two-thirds of women develop an isthmocele, defined as a hypoechoic defect at the uterotomy closure site on transvaginal ultrasound, within the first year after a first cesarean [8]. The niche is associated with postmenstrual spotting, dysmenorrhea, and chronic pelvic pain, and is the structural substrate for cesarean scar ectopic pregnancy and for placenta accreta spectrum in subsequent pregnancies [8]. An intervention applied at the time of the index operation that demonstrably reduces niche formation would carry consequences extending well beyond the immediate hospitalization.

Several randomized trials have examined PRP for cesarean wound healing over the past decade, each individually underpowered, with heterogeneous preparation protocols and outcome measures. The present systematic review and meta-analysis pools the available evidence, quantifies heterogeneity, addresses a methodological anomaly identified in one included study, and delineates what the current evidence base can and cannot support.

## Methods

This review was conducted and reported in accordance with the Preferred Reporting Items for Systematic Reviews and Meta-Analyses (PRISMA) 2020 statement [9]. The protocol was prospectively registered (INPLASY202640041; doi: 10.37766/inplasy2026.4.0041) [10].

### Eligibility criteria

We included studies in which women aged 18 years or older underwent cesarean section for any indication, the intervention was intraoperative PRP of either autologous maternal or umbilical cord blood origin, and the comparator was standard wound closure without PRP or with a placebo. Only randomized controlled trials were eligible. No restriction was placed on language or publication date. We excluded non-randomized designs, conference abstracts without extractable data, and studies covered by a published expression of concern or a retraction notice.

Primary outcomes were skin scar quality measured with POSAS, VSS, or the Manchester Scar Scale; early wound inflammation measured with the REEDA scale; and uterine scar integrity defined by niche formation rate and residual myometrial thickness (RMT). Secondary outcomes were postoperative pain measured by visual analogue scale (VAS) or numeric rating scale (NRS), analgesic consumption, wound complication rate, and quality of life.

The scales used to evaluate scarring differ in construction and interpretation, and their range and directionality are summarized here for clarity. POSAS is a validated dual-rater instrument: a patient component (six items, score range 6–60) captures the patient’s experience of the scar (pain, itch, colour, stiffness, thickness, irregularity), and an observer component (six items, score range 6–60) captures clinician judgment of vascularity, pigmentation, thickness, relief, pliability, and surface area; in both components a lower score indicates a better scar. The VSS evaluates pigmentation, vascularity, pliability, and height on a 0–13 scale, with a lower score again denoting a better outcome; the scale is widely used but has been criticized for limited inter-rater reliability in some settings. The REEDA scale assesses redness, edema, ecchymosis, discharge, and wound approximation in the early postoperative period, scoring 0–15 with lower scores reflecting less inflammation; it was developed for perineal episiotomy assessment and has subsequently been adopted for abdominal incisions, where it is most informative within the first one to two weeks. Where studies reported absolute POSAS scores that differed across cohorts, this reflects use of differing POSAS versions and total-score conventions; standardized mean differences were therefore the appropriate pooling metric.

### Search strategy

PubMed/MEDLINE, Embase, CENTRAL, Scopus, and Web of Science were searched from inception through March 2026. ClinicalTrials.gov and the WHO International Clinical Trials Registry Platform were searched for unpublished and ongoing trials. Reference lists of all included studies were hand-searched. The search string combined platelet-rich plasma terminology (“platelet-rich plasma”, “PRP”, “platelet rich plasma”, “cord blood platelet”, “platelet concentrate”) with cesarean delivery terminology (“cesarean”, “caesarean”, “C-section”) and wound or scar terminology (“wound healing”, “scar”, “uterine niche”, “isthmocele”, “surgical site infection”).

### Data extraction and statistical analysis

Two reviewers (S.Ş. and F.G.Ş.) extracted data independently; discrepancies were resolved by consensus or, where required, by a third reviewer. Continuous outcomes were summarized as standardized mean differences (Hedges’ g) with 95% confidence intervals using a DerSimonian–Laird random-effects model, with a negative SMD favouring PRP. Dichotomous outcomes were summarized as risk ratios with 95% confidence intervals. Heterogeneity was quantified using I² and the Cochran Q statistic; I² of 75% or higher triggered narrative synthesis rather than pooling. Two sensitivity analyses were pre-specified: an SD-versus-SEM interpretation for Thanachaiviwat 2024 (described in the next paragraph) and exclusion of studies rated as raising some concerns overall on risk of bias. Publication bias was to be assessed by funnel plot and Egger test where at least five studies contributed to a single pool; this threshold was not reached for any outcome. Evidence certainty was rated using the GRADE approach. Analyses were conducted in R 4.3.2 (metafor) and RevMan 5.4.

A dispersion-reporting ambiguity was identified in Thanachaiviwat 2024 [11] that warrants explicit description. Within a single results table, REEDA and VAS values carried “±” markers that, if treated as standard deviations, produced t-statistics of 13.8 (REEDA) and 3.7 (VAS) for *n* = 26 per group, inconsistent with the reported *p* = 0.009 and *p* = 0.473 respectively. When the values were instead treated as standard errors of the mean (so that SD = SEM × √n), the recalculated test statistics (t = 2.70, *p* ≈ 0.009; t = 0.72, *p* ≈ 0.473) were entirely consistent with the published p-values. In the same table, VSS values were internally consistent with the published *p* < 0.001 only when interpreted as standard deviations. The REEDA and VAS values for this study were therefore treated as SEMs throughout, and the full back-calculation is reported in Supplementary Table S1. The sensitivity analysis applying the naive standard-deviation interpretation is presented alongside the primary result.

### Risk of bias

Risk of bias was assessed using the Cochrane RoB 2 tool [12] across its five domains. S.Ş. and F.G.Ş. assessed each study independently. For Brezeanu 2025 [13], data were drawn exclusively from the registered randomized controlled trial (ClinicalTrials.gov NCT06978010; Healthcare 2025;13:2928). A concurrent single-arm observational study from the same group and centre (Healthcare 2025;13:2905) [14] was not included; it is cited only for completeness and dual-publication transparency.

## Results

### Study selection

**Fig. 1:**
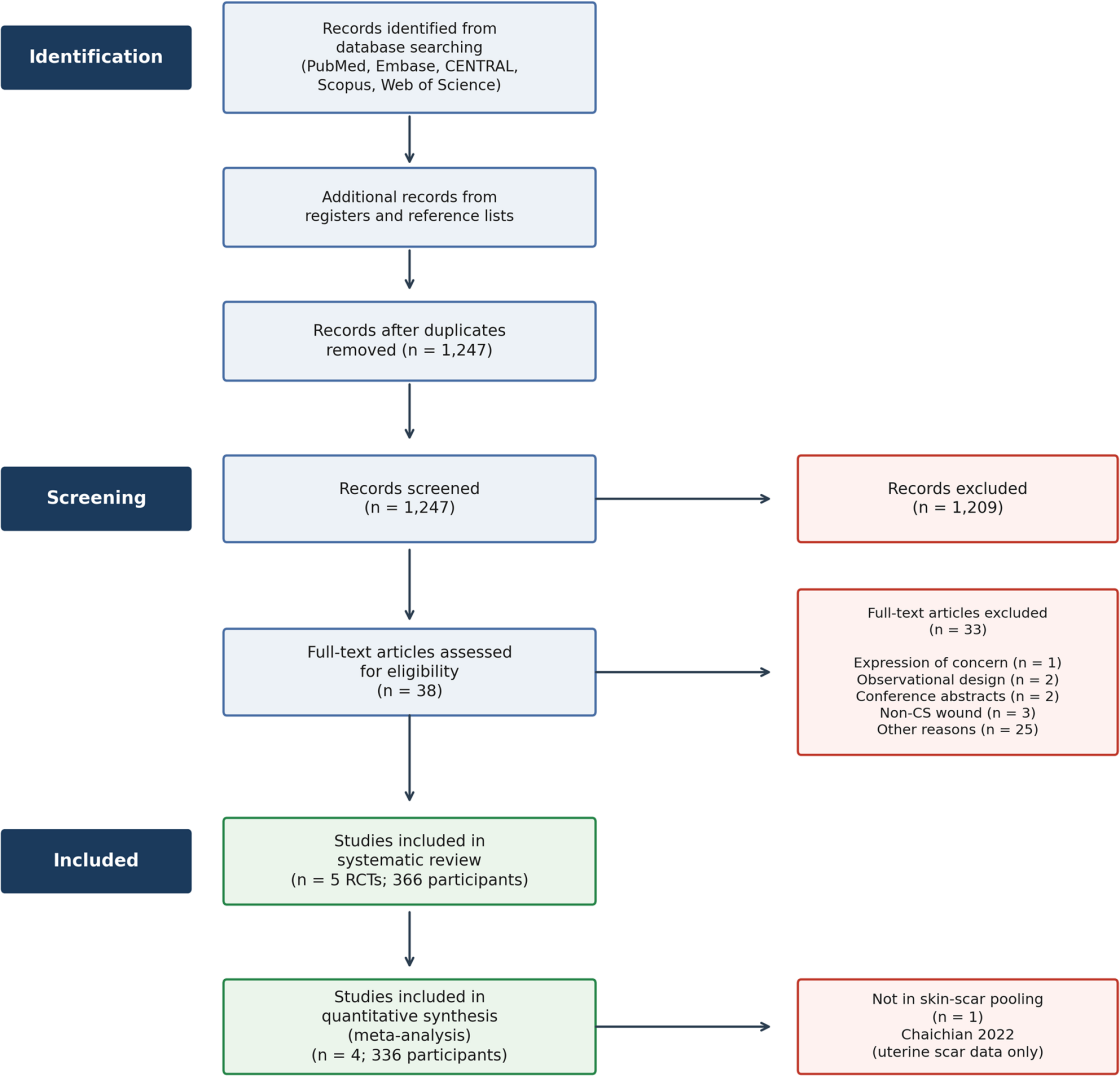
PRISMA 2020 flow diagram showing the study selection process. Database searches retrieved 1,247 unique records after deduplication. After title and abstract screening, 38 full-text articles were assessed; 33 were excluded. Five randomized trials (n = 366) were included in the systematic review, and four (n = 336) contributed to the quantitative synthesis of skin-scar outcomes

Database searches retrieved 1,247 unique records after deduplication. After title and abstract screening, 38 full-text articles were assessed for eligibility; 8 were excluded (expression of concern: Elkhouly 2021 [15]; observational design: *n* = 2; conference abstracts without extractable data: *n* = 2; non-cesarean wound: *n* = 3). Five randomized trials met the inclusion criteria and were retained for synthesis (Fig. 1).

### Study characteristics

The five included trials span five countries and a confirmed total of 366 women (Table 1). Two corrections from preliminary data extraction warrant explicit note. Tehranian 2016 [16] enrolled 67 women in the PRP arm and 71 in the control arm (total 138), rather than the *n* = 30 per arm attributed in several secondary citations. Chaichian 2022 [17] enrolled 15 women per arm (total 30) and evaluated the uterine scar rather than the abdominal skin: PRP was injected between the decidua and the myometrium at the time of uterotomy, and the primary outcomes were niche formation rate and residual myometrial thickness at six months. Chaichian 2022 is retained in the systematic review and contributes the uterine scar data, but is necessarily excluded from the skin-scar quantitative synthesis. Four trials (*n* = 336; 166 PRP, 170 control) contribute to skin-scar pooling.

**Table 1:** Characteristics of included randomized controlled trials

Study	*n* (PRP/Ctrl)	Population	PRP type	Application site	Primary outcome	Follow-up	Quant. synthesis
Tehranian 2016 [16] (Iran)	67/71 (138)	High-risk: DM, HTN, obesity	aPRP	Wound surface	REEDA, VAS	8 weeks	REEDA/VAS (narrative)
Chaichian 2022 [17] (Iran)	15/15 (30)	Elective CS	aPRP	Decidua–myometrium	Niche rate, RMT	6 months	Uterine scar only
Barwijuk 2024 [18] (Poland)	23/23 (46)	Low-risk, elective	aPRP (PDGFab 140 ng/mL)	Fascia + SC micro-injections	POSAS	90 days	POSAS, VAS
Thanachaiviwat 2024 [11] (Thailand)	26/26 (52)	Mixed (elective + emergency)	CB-PRP (cord blood, 2.5×)	SC (4 mL) + wound (1 mL)	REEDA, VSS	8 weeks	REEDA, VAS
Brezeanu 2025 [13] (Romania)¹	50/50 (100)	Low-risk, elective (BMI ≤ 35 kg/m²)	aPRP	Uterine margins (5 mL) + SC (5 mL)	POSAS	40 days	POSAS, REEDA, VSS, VAS

*aPRP *Autologous PRP, *CB-PRP* Cord blood PRP, *SC* Subcutaneous, *DM* Diabetes mellitus, *HTN* Hypertension, *RMT* Residual myometrial thickness

¹ Registered RCT (NCT06978010, Healthcare 2025;13:2928) only; concurrent observational study (Healthcare 2025;13:2905) [14] excluded

The five trials are also clinically heterogeneous to a degree that is essential to flag at the outset. Three of the five (Barwijuk 2024, Chaichian 2022, Brezeanu 2025) used autologous PRP prepared from maternal peripheral blood, with reported platelet concentration enrichment ranging from approximately 2.5-fold to 5-fold relative to baseline and with one trial (Barwijuk) reporting platelet-derived growth factor AB concentrations of 140 ng/mL. One trial (Thanachaiviwat 2024) used cord blood PRP collected from the umbilical vessels after delivery and concentrated approximately 2.5-fold, which is biologically distinct from maternal aPRP both in growth-factor profile and in donor population. The application site also differs across trials: subcutaneous administration alone, subcutaneous combined with fascial micro-injections, application at the uterine margins combined with subcutaneous administration, and injection between decidua and myometrium for the niche outcome. PRP dose varied from 1 to 10 mL. These differences in source, preparation, dose, and anatomic site mean that the included interventions, although all categorized as “intraoperative PRP,” are not directly comparable in a mechanistic sense, and pooled estimates should accordingly be interpreted as broad indicators of effect rather than as estimates of a single biologically uniform treatment.

### Risk of bias

Tehranian 2016 and Barwijuk 2024 were judged at low overall risk of bias. Thanachaiviwat 2024 was rated low overall, with domain 4 raising only the labelling concern described in Sect. Data extraction and statistical analysis ; the biological findings themselves are not in question. Chaichian 2022 raised some concerns for randomization reporting (domain 1) and selective outcome reporting (domain 5). Brezeanu 2025 raised some concerns for outcome measurement (domain 4: outcome assessors not blinded, although the statistician was). Detailed domain-level judgments are reported in Table 2.

**Table 2:** Risk of bias — Cochrane RoB 2 tool

Study	D1: Randomization	D2: Deviations	D3: Missing data	D4: Measurement	D5: Reporting	Overall
Tehranian 2016 [16]	Low	Low	Low	Low	Low	LOW
Chaichian 2022 [17]	Some concerns	Low	Some concerns	Low	Some concerns	SOME CONCERNS
Barwijuk 2024 [18]	Low	Low	Low	Low	Low	LOW
Thanachaiviwat 2024 [11]	Low	Low	Low	Some concerns (SEM labelling)	Low	LOW
Brezeanu 2025 [13]	Low	Low	Low	Some concerns (assessor blinding)	Low	SOME CONCERNS

### Quantitative results

**Fig. 2:**
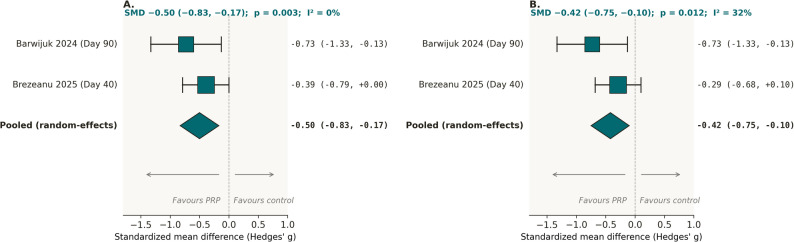
POSAS forest plots: patient component (**a**) and observer component (**b**). Patient SMD −0.50 (95% CI −0.83, −0.17), p = 0.003, I² = 0%; observer SMD −0.42 (95% CI −0.75, −0.10), p = 0.012, I² = 32%. Square size is proportional to inverse-variance weight; the diamond represents the pooled estimate. **A** POSAS - patient component. **B** POSAS - observer component

**Fig. 3:**
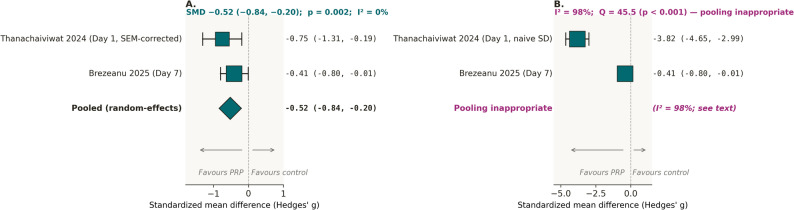
REEDA forest plot (days 1–7). Primary analysis using SEM-corrected values: SMD −0.52 (95% CI −0.84, −0.20), p = 0.002, I² = 0%. The dashed reference represents the sensitivity analysis under the naive SD assumption, in which I² reached 98% and pooling was invalid. **A** Primary analysis (SEM-corrected). **B** Sensitivity analysis (naive SD assumption)

**Fig. 4:**
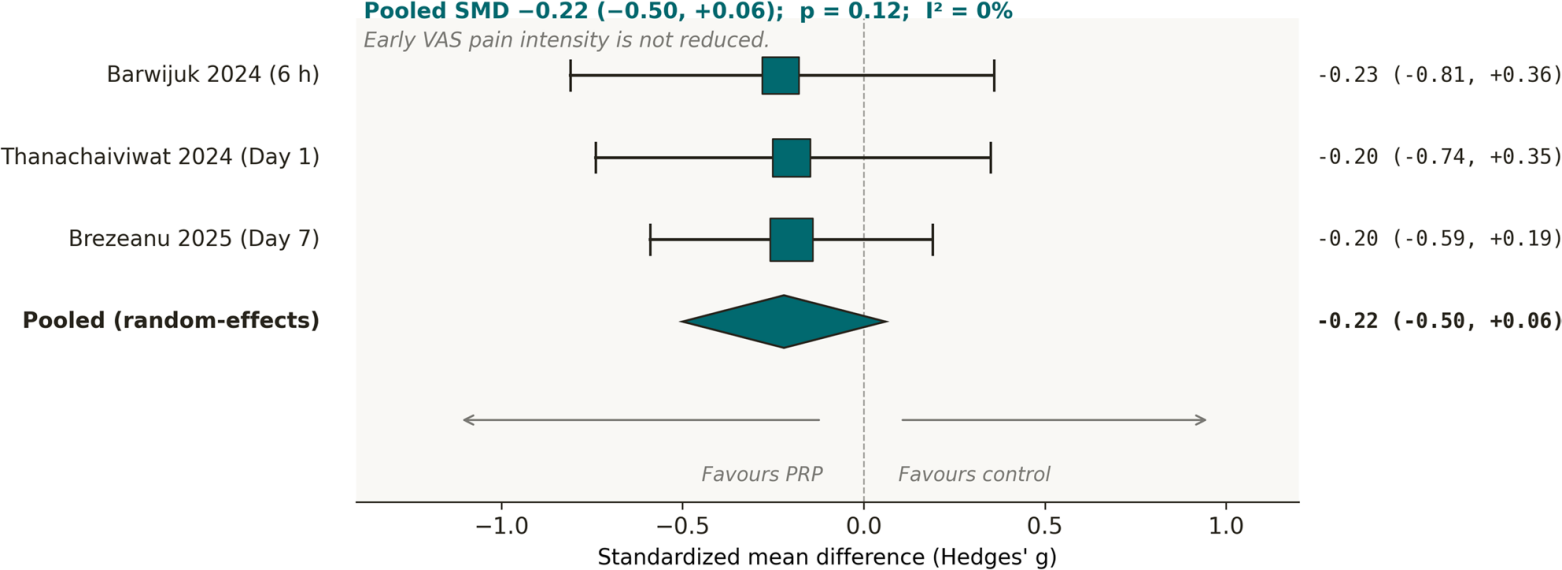
VAS pain intensity at early postoperative time points. Pooled SMD −0.22 (95% CI −0.50, +0.06), p = 0.12, I² = 0%. PRP does not appear to reduce early pain intensity

A consolidated summary of pooled estimates is presented in Table 3; forest plots for the principal outcomes are provided in Figs. 2, 3 and 4, and individual study-level data are in Table 4.

**Table 3:** Pooled meta-analysis estimates — DerSimonian–Laird random-effects model

Outcome	Studies	Patients (PRP/Ctrl)	Pooled effect (95% CI)	*p*	I²	Q (p_het)	GRADE
POSAS patient (40–90 days)	2	73/73	SMD − 0.50 (− 0.83, − 0.17)	0.003*	0%	Q = 0.87 (0.35)	Moderate¹
POSAS observer (40–90 days)	2	73/73	SMD − 0.42 (− 0.75, − 0.10)	0.012*	32%	Q = 1.47 (0.23)	Moderate¹
REEDA (days 1–7) [SEM-corrected†]	2	76/76	SMD − 0.52 (− 0.84, − 0.20)	0.002*	0%	Q = 0.96 (0.33)	Low–Moderate²
REEDA sensitivity (naive SD)	2	76/76	Cannot pool	—	98%	Q = 45.5 (< 0.001)	Cannot pool
Uterine niche rate (Chaichian 2022)	1	15/15	RR 0.29 (0.07, 1.16)‡	Fisher 0.109	N/A	—	Low³
VSS (weeks 6–8)	2	76/76	Cannot pool	—	94%	Q = 17.8 (< 0.001)	Narrative only
VAS pain (0–7 days)	3	99/99	SMD − 0.22 (− 0.50, + 0.06)	0.12	0%	Q = 0.10 (0.95)	Moderate¹
Analgesic consumption	2	53/53	Not poolable (different metrics)	—	—	—	Low²
Wound complication rate	5	≈ 186/195	0 events in all arms	—	—	—	Very Low³

* Statistically significant

† REEDA and VAS values for Thanachaiviwat 2024 treated as SEM; verified by p-value back-calculation (Supplementary Table S1)

‡ Calculated from the reported 2 × 2 Table (2/15 vs. 7/15); Fisher’s exact p = 0.109 (two-sided). The study-reported p = 0.002 is inconsistent with the binary niche rate and likely reflects a continuous endpoint (niche depth/height or RMT); see Sect. Uterine scar: Chaichian 2022 . GRADE-rating rationale

¹downgraded for imprecision (pool comprises two trials with modest aggregate sample size)

²downgraded for imprecision and indirectness (heterogeneous PRP preparations and predominantly low-risk populations)

³downgraded for imprecision and risk of bias (single small trial with internal statistical inconsistency)

**Table 4:** Extracted outcome data (mean ± SD unless stated)

Study	Outcome	Time point	Disp. unit	PRP mean ±	Control mean ±	*p*	SMD (95% CI)
Barwijuk 2024 [18] (*n* = 23/23)	POSAS pat.	Day 8	SD	16.96 ± 1.52	17.57 ± 1.85	0.230	−0.36 (− 0.94, + 0.22)
	POSAS pat.	Day 30	SD	17.00 ± 1.76	18.09 ± 2.00	0.033*	−0.58 (− 1.18, + 0.02)
	POSAS pat.	Day 90	SD	14.91 ± 1.54	16.09 ± 1.68	0.021*	−0.73 (− 1.33, − 0.13)
	POSAS obs.	Day 90	SD	13.39 ± 1.53	14.74 ± 2.11	0.002*	−0.73 (− 1.33, − 0.13)
	VAS	0 h	SD	3.30 ± 2.23	3.96 ± 2.08	0.299	−0.31 (− 0.89, + 0.28)
	VAS	6 h	SD	4.96 ± 1.49	5.30 ± 1.52	0.465	−0.23 (− 0.81, + 0.36)
Thanachaiviwat 2024 [11] (*n* = 26/26)	REEDA	Day 1	SEM†	1.50 ± 0.256	2.50 ± 0.267	0.009*	−0.75 (− 1.31, − 0.19)
	REEDA	Day 3	SEM†	0.92 ± 2.60	1.12 ± 2.17	0.572	−0.08 (− 0.63, + 0.47)
	VSS	Week 8	SD	2.58 ± 2.00	6.96 ± 2.44	< 0.001*	−1.97 (− 2.63, − 1.30)
	VAS	Day 1	SEM†	3.73 ± 0.439	4.19 ± 0.464	0.473	−0.20 (− 0.74, + 0.35)
Chaichian 2022 [17] (*n* = 15/15)	Niche rate	6 months	binary %	2/15 (13.3%)	7/15 (46.7%)	Fisher *p* = 0.109‡	RR 0.29 (0.07, 1.16)
Brezeanu 2025 [14] (*n* = 50/50)	POSAS pat.	Day 7	SD	10.08 ± 3.28	11.00 ± 4.00	0.231	−0.25 (− 0.65, + 0.14)
	POSAS pat.	Day 40	SD	7.24 ± 1.81	8.00 ± 2.06	0.029*	−0.39 (− 0.79, + 0.00)
	POSAS obs.	Day 40	SD	6.46 ± 1.23	6.84 ± 1.39	0.300	−0.29 (− 0.68, + 0.10)
	REEDA	Day 7	SD	0.88 ± 1.09	1.56 ± 2.10	NS	−0.41 (− 0.80, − 0.01)
	VSS	Day 40	SD	0.46 ± 0.73	0.76 ± 1.18	NS	−0.31 (− 0.70, + 0.09)
	VAS	Day 7	SD	1.08 ± 0.89	1.30 ± 1.32	NS	−0.20 (− 0.59, + 0.19)
	NRS	Day 40	SD	0.10 ± 0.36	0.60 ± 0.80	p not reported (CI excludes null)	−0.81 (− 1.21, − 0.40)

The NRS day-40 entry, although not reported as significant in the original study, has a 95% confidence interval that excludes the null, so an exact p is unavailable but the effect estimate is internally consistent with a meaningful difference

*NRS* Numeric Rating Scale, *NS* Not significant in the original report (exact p value not given)

* *p* < 0.05

† Reported as SEM; SD used in analysis (SD = SEM × √26; Supplementary Table S1)

‡ Fisher’s exact p calculated from 2×2 table; study-reported p = 0.002 is inconsistent with this table and likely reflects a continuous niche dimension or RMT

#### Skin scar quality: POSAS patient

Two trials contributed patient-reported scar data at follow-up beyond one month. Barwijuk 2024 [18] reported a day-90 mean of 14.91 ± 1.54 in the PRP arm and 16.09 ± 1.68 in the control arm (SMD − 0.73, 95% CI − 1.33 to − 0.13), and Brezeanu 2025 [13] reported a day-40 mean of 7.24 ± 1.81 versus 8.00 ± 2.06 (SMD − 0.39, 95% CI − 0.79 to 0.00). The absolute scores differ between trials because the two studies use different POSAS total-score conventions; SMD pooling is the appropriate metric for this reason. The pooled estimate was SMD − 0.50 (95% CI − 0.83 to − 0.17; *p* = 0.003; I² = 0%; Fig. 2a). No wound dehiscence was observed in either arm of either trial.

#### Skin scar quality: POSAS observer

The same two trials contributed observer-rated data. Barwijuk 2024 reported 13.39 ± 1.53 versus 14.74 ± 2.11 at day 90 (SMD − 0.73), and Brezeanu 2025 reported 6.46 ± 1.23 versus 6.84 ± 1.39 at day 40 (SMD − 0.29). The pooled estimate was SMD − 0.42 (95% CI − 0.75 to − 0.10; *p* = 0.012; I² = 32%; Fig. 2b). The moderate heterogeneity is directionally consistent across studies; the smaller observer effect in Brezeanu at day 40 is biologically coherent, as the inflammatory signs that dominate clinician assessment resolve more slowly than the scar characteristics that patients report.

#### Early wound inflammation: REEDA

Two trials contributed early REEDA data. Thanachaiviwat 2024 reported a day-1 mean of 1.50 versus 2.50 in the CB-PRP and control arms respectively, treated as SEM after the back-calculation described in Sect. Data extraction and statistical analysis (recalculated SD = 1.31 in each arm; individual SMD − 0.75, 95% CI − 1.31 to − 0.19). Brezeanu 2025 reported a day-7 mean of 0.88 ± 1.09 versus 1.56 ± 2.10 (SMD − 0.41, 95% CI − 0.80 to − 0.01). The pooled estimate was SMD − 0.52 (95% CI − 0.84 to − 0.20; *p* = 0.002; I² = 0%; Fig. 3). The sensitivity analysis under the naive standard-deviation interpretation produced an individual SMD of − 3.82 for Thanachaiviwat and an I² of 98% with Brezeanu; under that assumption, pooling is inappropriate. The primary result is accordingly conditional on the SEM identification, which the internal back-calculation supports.

#### Uterine scar: Chaichian 2022

A single small randomized trial (*n* = 15 per arm) examined the uterine scar. PRP was injected between the decidua and the myometrium at uterotomy closure. At six-month transvaginal ultrasound, niche formation was observed in 2 of 15 PRP patients (13.3%) and in 7 of 15 control patients (46.7%), giving a risk ratio of 0.29 (95% CI 0.07 to 1.16) and a Fisher’s exact two-sided p-value of 0.109 calculated from the published 2 × 2 table.

The study-reported p-value of 0.002 for this comparison could not be reproduced from the published binary table by any standard test: Fisher’s exact gives *p* = 0.109, and chi-square without continuity correction gives *p* = 0.046. The most plausible explanation is that the *p* = 0.002 derives from a continuous endpoint reported within the same table — niche depth, niche height, or residual myometrial thickness — rather than from the binary occurrence of niche. The binary result therefore does not reach conventional statistical significance by any extraction-based test; the absolute 33-percentage-point difference is nonetheless large and directionally consistent with the biological hypothesis. We treat this finding as hypothesis-generating, and consider that a confirmatory trial with adequate sample size and pre-specified continuous niche endpoints is the appropriate next step.

#### Vancouver scar scale

The two trials reporting VSS were not pooled because the difference between their estimates exceeded the I² ≥ 75% threshold pre-specified for narrative reporting. Thanachaiviwat 2024 (CB-PRP, week 8) reported 2.58 ± 2.00 versus 6.96 ± 2.44 (SMD − 1.97, 95% CI − 2.63 to − 1.30; *p* < 0.001), whereas Brezeanu 2025 (autologous PRP, day 40) reported 0.46 ± 0.73 versus 0.76 ± 1.18 (SMD − 0.31, 95% CI − 0.70 to + 0.09; p = NS). The I² of 94% (Q = 17.8, *p* < 0.001) reflects differences in PRP source, population, anaesthesia, follow-up duration, and scoring habits, and a pooled VSS estimate would obscure rather than clarify the underlying picture.

#### Pain and analgesic consumption

Three trials contributed VAS pain data at early time points. The pooled estimate was SMD − 0.22 (95% CI − 0.50 to + 0.06; *p* = 0.12; I² = 0%; Fig. 4); early pain intensity was not reduced. Analgesic consumption gave a different picture in the two trials that reported it: in Barwijuk 2024 the PRP arm required fewer paracetamol doses per day (*p* = 0.006) and only one PRP patient required supplemental ketoprofen compared with six controls, and in Tehranian 2016 a 93% cumulative VAS reduction was reported in the PRP arm versus 79% in controls at eight weeks (*p* < 0.001). The apparent discrepancy between early pain scores and analgesic patterns is not contradictory: a 6-hour visual analogue score in the recovery room captures a different physiological moment than the pattern of drug use across several days of recovery.

#### Individual study data

## Discussion

The principal finding of this synthesis is that intraoperative platelet-rich plasma, applied to the cesarean wound at the time of surgery, improves skin scar quality by approximately 0.4 to 0.5 standard deviations on both patient and clinician assessment at follow-up between 40 and 90 days. The patient-reported component of POSAS is the most stable signal in the analysis: two trials carried out in different populations, with different PRP preparations and different follow-up durations, produced nearly identical effect estimates and zero residual heterogeneity (I² = 0%), and neither study observed wound dehiscence in either arm. The biological reading is straightforward in this case; the patient experiences the scar across pain, itch, colour, stiffness, thickness, and irregularity, and across two independent trials this composite experience is consistently more favourable when PRP has been applied at the time of closure.

The observer-rated POSAS signal is smaller and more heterogeneous (SMD − 0.42, I² = 32%), with the divergence plausibly driven by the earlier 40-day follow-up of Brezeanu 2025, since clinician judgment depends partly on inflammatory features (vascularity, pigmentation) that resolve more slowly than the scar characteristics patients perceive. Early wound inflammation, indexed by REEDA, is also reduced in the same direction; this finding is conditional on the dispersion-measure identification discussed in the next section, but once that identification is made the two contributing trials are internally consistent. Taken together, the POSAS-observer and REEDA findings fit a biologically coherent pattern in which PRP accelerates structural reorganization of healing tissue in a way that becomes detectable as a scar difference at six to twelve weeks rather than as an acute change in the first postoperative week.

The pain story is more nuanced. Early visual-analogue pain intensity at 0 to 7 days is not significantly reduced (pooled SMD − 0.22, *p* = 0.12), yet analgesic consumption is lower in the two trials that reported it: Barwijuk 2024 reported fewer paracetamol doses per day and fewer patients requiring supplemental ketoprofen in the PRP arm, and Tehranian 2016 reported a substantially larger cumulative VAS reduction in the PRP arm at eight weeks. The Brezeanu 2025 NRS measurement at day 40 (SMD − 0.81, 95% CI − 1.21 to − 0.40) is similarly directionally favourable. These observations are not contradictory: a 6-hour pain score in the recovery room captures peak acute nociception, whereas the pattern of drug use across several days of recovery and the residual pain perceived at six weeks reflect different physiological processes. PRP appears not to blunt the acute nociceptive signal of the first day or two after surgery, while contributing to a more comfortable recovery trajectory thereafter.

The uterine scar deserves separate treatment. We initiated this review expecting to describe uterine scar integrity as a research gap that the meta-analytic literature had not addressed. The gap is in fact narrower than expected, because Chaichian 2022 [17] enrolled fifteen women per arm, injected PRP between decidua and myometrium at uterotomy closure, and reported niche formation in 13.3% of PRP and 46.7% of control patients at six months. The absolute difference of 33% points is striking, but three constraints temper its interpretation. First, the sample size of thirty patients in total is small for any binary outcome with this base rate. Second, the published p-value of 0.002 cannot be reproduced from the reported 2 × 2 table by any standard binary test, and almost certainly reflects analysis of a continuous endpoint (niche depth, niche height, or RMT) that appears in the same table. Third, the Fisher’s exact p calculated from the binary data we can extract is 0.109, which does not reach conventional statistical thresholds. The clinical context — niche formation in nearly half of low-risk elective cesareans, with documented consequences for menstrual symptoms, scar pregnancy, and accreta spectrum in subsequent pregnancies — makes the signal worth pursuing, but the appropriate response is replication in an adequately powered trial with pre-specified continuous niche endpoints, not adoption into practice. We therefore treat this finding throughout as preliminary and hypothesis-generating.

The dispersion-reporting issue in Thanachaiviwat 2024 deserves brief comment because it illustrates a problem that recurs more widely in the surgical wound-healing literature. The back-calculation we describe in Sect. Data extraction and statistical analysis is not technically complex; it consists of asking whether the published test statistics are recoverable from the published means and reported dispersion units. When that check is not performed, REEDA values treated as standard deviations yield an individual standardized mean difference of − 3.82, an I² of 98% with Brezeanu 2025, and the correct but spurious conclusion that pooling is inappropriate. The sensitivity analysis in Table 3 illustrates precisely this scenario. The practical implication is that dispersion measures in clinical trial tables should be explicitly labelled as SD, SEM, or 95% confidence interval, meta-analysts should verify these labels against reported test statistics before pooling, and journals should require explicit dispersion-unit labelling as a condition of publication.

The contrast between Thanachaiviwat 2024 and Brezeanu 2025 on the Vancouver Scar Scale (individual SMDs of − 1.97 versus − 0.31, with I² of 94%) is too large to be reduced to a single number through pooling. Cord blood PRP delivers a higher concentration of several key growth factors than adult peripheral blood [7], and a real difference in efficacy between sources is biologically plausible. However, the two trials also differed in population, anaesthesia technique, centre, follow-up interval, and assessor habits, so the source effect cannot be isolated in the available data. Disentangling the PRP-source signal from these covariates would require a head-to-head three-arm trial that does not yet exist.

A further limitation that bears emphasis is that the patient population studied in most of the included trials is not the patient population in whom wound complications carry the greatest absolute burden. Three of five included trials excluded women with diabetes, body mass index above 35 to 40 kg/m², or emergency cesarean — the precise groups in whom wound infection, dehiscence, and impaired healing are most common. Tehranian 2016, the trial that did enrol this high-risk population, produced the most substantial REEDA improvement and is the only trial in our synthesis with clinically meaningful complication rates against which a benefit could be measured. The remaining trials enrolled healthy young women undergoing planned cesareans for breech presentation or maternal request, a group in whom wound complication rates are in the single digits regardless of intervention. A demonstration of PRP efficacy in that population is biologically informative; a demonstration in obese, diabetic, or repeat-cesarean patients is what would change clinical practice.

The overall evidence base supporting our conclusions is therefore both small and clinically heterogeneous. Five randomized trials, most outcome pools containing only two trials, per-arm sample sizes ranging from 15 to 71, three distinct categories of PRP preparation, four different anatomic application sites, and a predominance of low-risk elective populations together imply that the pooled estimates we present should be read as broad indicators of direction and magnitude rather than as precise quantifications of a single uniform treatment effect. Skin scar follow-up was capped at 90 days; longer-term scar evolution remains unmeasured. The uterine scar evidence rests on a single trial of thirty patients. The REEDA pooling is conditional on dispersion-measure identification. With fewer than five trials contributing to any single pool, formal publication-bias assessment was not possible. GRADE certainty is moderate for the POSAS outcomes, low to moderate for REEDA, and low for the uterine outcomes. The most useful next steps are accordingly clear: a multicentre randomized trial of intraoperative PRP in high-risk cesarean patients (obesity, diabetes, emergency cesarean, repeat cesarean), a head-to-head comparison of cord blood and autologous PRP within a single protocol, and an adequately powered trial with uterine scar integrity, measured by both binary niche formation and continuous niche dimensions, as a primary endpoint.

## Conclusions

Across five randomized trials enrolling 366 women, intraoperative PRP applied to the cesarean wound consistently improves skin scar quality on both patient and clinician assessment (POSAS-patient SMD − 0.50, *p* = 0.003, I² = 0%; POSAS-observer SMD − 0.42, *p* = 0.012, I² = 32%) and reduces early wound inflammation (REEDA SMD − 0.52, *p* = 0.002, I² = 0%), while early pain intensity is not reduced. A single small randomized trial provides the first randomized evidence that PRP applied directly to the uterine incision may reduce isthmocele formation, but with thirty patients in total, an unreproducible study-reported p-value, and a Fisher’s exact p of 0.109 on the binary outcome, this signal is hypothesis-generating and requires prospective replication before any change in practice. The available trials enrolled predominantly low-risk elective cesarean populations and used heterogeneous PRP preparations, dose, and application sites; the pooled estimates should therefore be read as indicators of effect direction rather than as precise treatment quantifications. Three trials in particular are needed before PRP can be recommended for routine use: a multicentre trial in high-risk obstetric populations, a head-to-head trial comparing cord blood and autologous PRP, and an adequately powered uterine-scar trial with pre-specified continuous niche endpoints.

## Supplementary Information

Supplementary Material 1

## Data Availability

All data generated or analysed during this study are included in this published article and its supplementary information files. Extracted data are presented in Tables 1, 2, 3 and 4 and Supplementary Table S1.

## Abbreviations

PRP: Platelet-rich plasma
aPRP: Autologous platelet-rich plasma
CB-PRP: Cord blood platelet-rich plasma
CS: Cesarean section
POSAS: Patient and Observer Scar Assessment Scale
REEDA: Redness, Edema, Ecchymosis, Discharge, Approximation scale
VSS: Vancouver Scar Scale
VAS: Visual Analogue Scale
NRS: Numeric Rating Scale
RCT: Randomized controlled trial
SMD: Standardized mean difference
MD: Mean difference
RR: Risk ratio
CI: Confidence interval
SD: Standard deviation
SEM: Standard error of the mean
RoB 2: Cochrane Risk of Bias 2 tool
GRADE: Grading of Recommendations Assessment, Development and Evaluation
PRISMA: Preferred Reporting Items for Systematic Reviews and Meta-Analyses
RMT: Residual myometrial thickness
ERAS: Enhanced Recovery After Surgery
BMI: Body mass index
