# Supplementary material for: Platelet-rich plasma for wound healing and scar formation after cesarean section: a systematic review and meta-analysis of randomized controlled trials
Source: BMC Pregnancy Childbirth. 2026 Jun 11;26:906. doi: 10.1186/s12884-026-09447-w (PMC13479406; doi:10.1186/s12884-026-09447-w)
Supplement: Supplementary file 1 [file 12884_2026_9447_MOESM1_ESM.docx]

**Supplementary Table S1. Dispersion Measure Verification — Thanachaiviwat 2024 (n=26/group)**

Formula: t = |m₁ − m₂| / (Sp × √[2/n]); df=50; two-sided p from t-distribution. An incompatible t identifies the wrong dispersion assumption.

***Supplementary Table S1. Back-calculation verification***

| **Outcome** | **Reported ±** | **Assumption** | **Pooled SD** **(computed)** | **t** | **p** | **Verdict** |
| --- | --- | --- | --- | --- | --- | --- |
| REEDA day 1 (1.5 vs 2.5, p=0.009) | 0.256/0.267 | Treat as SD | 0.262 | 13.78 | <0.001 | WRONG ❌ |
| REEDA day 1 | 0.256/0.267 | SEM → SD (×√26) | 1.333 | 2.70 | 0.009 | CORRECT ✔ |
| VAS day 1 (3.73 vs 4.19, p=0.473) | 0.439/0.464 | Treat as SD | 0.452 | 3.68 | <0.001 | WRONG ❌ |
| VAS day 1 | 0.439/0.464 | SEM → SD (×√26) | 2.302 | 0.72 | 0.473 | CORRECT ✔ |
| VSS week 8 (2.58 vs 6.96, p<0.001) | 2.003/2.441 | Treat as SD | 2.233 | 7.08 | <0.001 | CORRECT ✔ |
| VSS week 8 | 2.003/2.441 | SEM → SD (×√26) | 11.39 | 1.39 | 0.17 | WRONG ❌ |

*❌=contradicts reported p; ✔=consistent. REEDA and VAS use SEM; VSS uses SD. All three are reported as unlabelled ± values in the same table of the original paper.*
